# Promising Results of Kidney Transplantation From Donors Following Euthanasia During 10-Year Follow-Up: A Nationwide Cohort Study

**DOI:** 10.3389/ti.2024.13142

**Published:** 2024-10-18

**Authors:** Charlotte Susanna, Nathalie van Dijk, Wim de Jongh, Hanne Verberght, Walther van Mook, Jan Bollen, Bas van Bussel

**Affiliations:** ^1^ Department of Intensive Care Medicine, Maastricht University Medical Center+, Maastricht, Netherlands; ^2^ Heart and Vascular Center, Maastricht University Medical Center+, Maastricht, Netherlands; ^3^ Department of Surgery, Maastricht University Medical Center+, Maastricht, Netherlands; ^4^ School of Nutrition and Translational Research in Metabolism, Faculty of Health, Medicine and Life Sciences, Maastricht University, Maastricht, Netherlands; ^5^ Academy for Postgraduate Medical Training, Maastricht University Medical Center+, Maastricht, Netherlands; ^6^ School of Health Professions Education, Faculty of Health, Medicine and Life Sciences, Maastricht University, Maastricht, Netherlands; ^7^ Department of Anesthesiology, Pain and Palliative Medicine, Radboud University Medical Center, Nijmegen, Netherlands; ^8^ Care and Public Health Research institute (Caphri), Faculty of Health, Medicine and Life Sciences, Maastricht University, Maastricht, Netherlands; ^9^ Cardiovascular research institute Maastricht (Carim), Faculty of Health, Medicine and Life Sciences, Maastricht University, Maastricht, Netherlands

**Keywords:** organ donation, euthanasia, donation after circulatory death, donation after brain death, kidney transplantation, organdonation after euthanasia, medical assistance in dying, physician assisted death

## Abstract

The outcome of kidneys transplanted following organ donation after euthanasia (ODE) remains unclear. This study analyzed all kidney transplantations in the Netherlands from January 2012 to December 2021, comparing the outcomes following ODE, donation after circulatory death (DCD-III), and donation after brain death (DBD). 9,208 kidney transplantations were performed: 148 ODE, 2118 DCD-III, and 1845 DBD. Initial graft function was compared between these categories. Immediate graft function, delayed graft function and primary non-function in ODE kidney recipients were 76%, 22%, and 2%, respectively, 47%, 50% and 3% in DCD-III kidney recipients and 73%, 25%, and 2% in DBD kidney recipients (overall p-value: p < 0.001). The number of kidneys transplanted over a median follow-up period of 4.0 years (IQR 2.0–6.6), was 1810, including 72 ODE, 958 DCD-III and 780 DBD kidneys. In this period, 213 grafts (11.8%) failed [7 grafts (9.7%) from ODE donors, 93 grafts (9.7%) from DCD-III donors, and 113 grafts (14.5%) from DBD donors]. Kidneys transplanted after euthanasia have a good immediate graft function, a comparable longitudinal 10 years eGFR, and similar graft failure hazard to kidneys from DCD-III and DBD. Kidney transplantation following ODE is a valuable and safe contribution to the donor pool.

## Graphical Abstract

**Figure:**
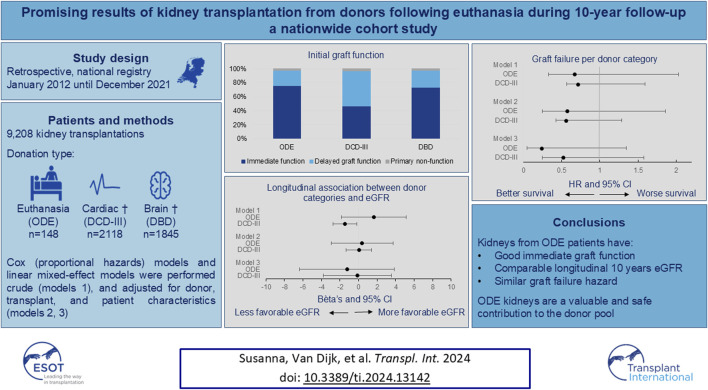

## Introduction

Post-mortem organ donation can be performed after brain death (Donation after Brain Death, DBD) or following circulatory death (Donation after Circulatory Death, DCD). DCD is categorized based on the Maastricht classification into four types, of which DCD-I, DCD-II, and DCD-IV are classified as uncontrolled donations; DCD-III is classified as donation following withdrawal of life-sustaining therapy and is a controlled donation [1]. Since July 2017, DCD-I and DCD-II procedures are no longer performed in the Netherlands.

In ODE, a patient dies in a controlled manner, following administration by a physician of euthanasia drugs. However, the dying process of these patients differs from that of patients who donate their organs after circulatory death (i.e., DCD) following withdrawal of life-sustaining treatments in the ICU (i.e., DCD-III) or following brain death (i.e., DBD), as these patients are critically ill [2]. Hence, the DCD classification recently proposed to include ODE patients in a separate category of highly controllable DCD: DCD-V [3, 4].

As the present study describes data from Netherlands, we use organ donation after *euthanasia* (ODE) as terminology rather than MAID (Medical Assistance in Dying), which is used in other countries [5, 6]. The ethical, legal, and logistical implications of ODE, in general, have been extensively discussed in both the scientific literature and public media [7, 8].

The possibility of ODE is expanding to more countries, and the number of procedures is increasing annually in most countries where ODE is already available [9, 10]. Organ donation after euthanasia may increase the number of donor organs and thus aid in narrowing the gap between the demand and availability of organs for transplantation. The next question to consider is whether the outcomes of the transplanted organs after ODE are sufficient to continue the procedure.

Data on the outcome of kidneys transplanted following ODE is scarce [11]. A conference abstract reported graft function of transplanted kidneys following ODE that was comparable to DCD-III and DBD over a 5-year follow-up period [12]. We hypothesized that transplant outcomes after ODE have favorable initial graft function, favorable estimated glomerular filtration rate, and less graft failure compared to transplant outcomes from DCD-III and DBD over a 10-year follow-up period. We investigated whether this was independent of a comprehensive set of donor, recipient, and transplant variables. This investigation provides the results of kidney transplants following ODE compared to kidney transplants from other forms of donation during a 10-year study period.

## Patients and Methods

Data from the Dutch Transplant Foundation (Nederlandse Transplantatie Stichting, NTS) are recorded in the Netherlands Organ Transplantation Registry (NOTR), which includes all kidney transplantations performed in the Netherlands. The authors requested and obtained data on transplantations between 1st January 2012 (the year of the first ODE retrieval in the Netherlands), and 31st December 2021, from the NTS registry NOTR in accordance with their data registry governance.

These data were used to construct a retrospective cohort of patients who underwent a kidney transplantation, to compare the graft function between ODE, DCD-III, and DBD derived grafts.

Next, we excluded organ transplantations from donors younger than 18 years, and DCD-I, DCD-II, and DCD-IV donations, as defined in the Maastricht Category [1], and living donation retrievals. No donors in the dataset were represented in multiple transplantation categories (e.g., a living transplantation followed by a post-mortem donation). This resulted in the following categories to be studied: organ donation after euthanasia (ODE, DCD-V); organ donation after circulatory death, Maastricht Category III (DCD-III); and organ donation after brain death (DBD) [1, 3].

Recipients may undergo multiple kidney transplantations during their disease course. For the primary investigation, we included and characterized the most recent transplant (i.e., the latest transplant) in a recipient. In this way any recipient with multiple kidney transplantations was included only once in the analyses.

We described the donors, the recipients, and transplantation and graft characteristics for ODE (DCD-V), DCD-III and DBD categories. Initial graft function, as well as estimated glomerular filtration (eGFR) rate over 10 years and graft failure, were described for ODE, DCD-III and DBD categories.

### Donor Characteristics

For donor characteristics, we described age in years, sex, serum creatinine concentrations in µmol/L, medical history of hypertension, diabetes mellitus, and smoking status (dichotomous outcome measures and in pack years), as reported in the NOTR.

### Recipient Characteristics

For recipient characteristics, age was defined as the recipient’s age at transplantation in years. Furthermore, we described sex, dialysis time and panel reactive antibody (PRA). The PRA test was used to estimate the degree of sensitization in recipients’ blood to donor-specific antibodies. Traditionally, the recipient’s serum is exposed to a panel of random donor lymphocytes. The PRA test indicates the risk of transplant failure to the host response to transplantation [13, 14]. PRA is classified as low (≤5%), intermediate (6%–84%), and high (≥85%). Dialysis time was measured as the days of dialysis of the recipient before transplantation and presented in years by dividing by 365.25.

### Transplantation Characteristics

The warm ischemia time (WIT) is defined as the time between the circulatory arrest (e.g., loss of cardiac output in a DCD-III and ODE (DCD-V), and arterial clamping in DBD until the start of cold aortic flush (*in situ* preservation) or the start of normothermic aortic flush in case of normothermic machine perfusion of the donor [15]. The cold ischemia time (CIT) is defined as the start of cold aortic flush (*in situ* preservation) until cessation of hypothermic machine perfusion respectively taken off ice. The anastomosis time (AT) is defined as the time between the end of the hypothermic state and reperfusion of the kidney in the recipient.

### Graft Characteristics

#### Initial Graft Function

Graft function within the first week post-transplantation was categorized into primary non-function, delayed graft function, and immediate graft function. Kidney transplantations that failed (e.g., non-viable kidneys, or graft loss) in the first week post-transplantation were categorized as primary non-function. Kidney transplantations that required dialysis the first week post-transplantation were categorized as delayed graft function. The remaining kidney transplantations were categorized as immediate graft function.

#### Graft Failure

Graft failure and its causes were pre-scored in the NOTR and included hyperacute rejection, infection (not graft-related), infection of graft, non-viable kidney, patient dying with a functioning transplant, permanent non-function, recurrent primary renal disease, rejection after stopping all immunosuppressive drugs, rejection while taking immunosuppressive drugs (acute/chronic), removal of functioning graft, technical problems, thrombosis/infarction, vascular or ureteric problems, vascular problems: none-operative or rejection related, other (renal) and unknown (Supplementary Table S1).

#### Estimated Glomerular Filtration Rate

Graft function was studied using 10-year follow-up on serum creatinine. The eGFR was calculated using the re-expressed MDRD-4-formula [16]. The concentration of serum creatinine (in µmol/L) was converted to serum creatinine in mg/dL by using the molecular weight of creatinine (113.12 g/mol). Increased age of recipients for creatinine measurements post-transplant was considered (e.g., for eGFR estimation 2 years post-transplant, the following age was used: age at date of transplantation plus 2 × 365.25). The eGFR was presented as mean and standard error (SE) to indicate that time moments may have more or fewer observations in the ODE, DCD-III, and DBD groups. Invalid (negative) creatinine values were removed from the dataset (n = 1,021). The number of invalid creatinine values at 3 months, 1 year, 2 years, 3 years, 4 years, 5 years, 6 years, 7 years, 8 years, 9 years, and 10 years were, respectively, 42, 36, 110, 178, 167, 140, 111, 102, 67, 43, and 25.

### Statistical Analysis

This observational study is reported in accordance with the STROBE guideline [17].

Continuous data was visually inspected for normality and presented as mean ± standard deviation or as median (interquartile range). Categorical variables were presented as percentages. One-way ANOVA, Chi-square test, and Fisher’s exact test were used to test overall differences between ODE, DCD-III and DBD categories. Pairwise comparisons were conducted as post-hoc analysis to identify differences between 2 of the 3 categories if an overall test indicated statistical significance.

First, we used linear mixed-effects models to analyze whether longitudinal kidney function over 10 years, based on eGFR, differed between donor categories, with DBD as the reference category. We investigated a model containing donor category and time as independent variables (model 1). Model 1 was subsequently adjusted for donor age, donor sex, donor smoking, recipient age, recipient sex, and transplant ischemic times (CIT and AT) (model 2). Next, this model was further adjusted for donor hypertension, donor diabetes mellitus, WIT and transplant PRA (model 3). Recipient ID was added as a random effect to the models. The longitudinal mixed-effects models were repeated with time as random slopes. Fixed effects were presented as coefficients (β) and 95% CI, with a negative coefficient indicating a lower eGFR per donor category, as compared to the reference category.

Then, we used Cox proportional hazard models for the main analyses to investigate the association between donor categories and graft failure, with DBD as reference category. Grafts in which primary non-function occurred within the first week post-transplant, and therefore failing grafts, were excluded from the primary Cox analyses, because it is considered a short-term outcome with another presumed mechanism than those involved over the longer periods of time. Death of the recipient was considered a censored event in the main analyses when the recipient died with a functioning graft. Crude (model 1) models were adjusted (models 2 and 3) for the same set of variables in accordance with adjustments for the linear-mixed effects models above. For the Cox models, we report hazard ratios (HR) with their 95% confidence intervals (CI), with an HR higher than 1 indicating an increased hazard per donor category as compared to the reference category. The proportional hazards assumption was checked using the scaled Schoenfeld residuals.

We performed four sensitivity analyses and re-analyzed the above Cox models by first replacing the recipients for organs donated (i.e., including all transplantations of each recipient; sensitivity analysis 1) to determine whether the outcomes remain consistent with the primary models. Next, we also re-analyzed model 1 and model 2, in which recipient death was not censored, but included as an event (sensitivity analysis 2). A third sensitivity analysis was performed to re-analyze model 1 and model 2, in which primary non-function, which was assumed to have a separate etiology from graft failure occurring after a prolonged period, was included (sensitivity analysis 3). Finally, a fourth sensitivity analysis was performed re-analyzing model 1 and model 2, in which only the first transplantation within a recipient was used in the analyses, instead of the last transplantation within a recipient in the primary analyses. Although we assumed that matching a kidney between the donor and recipient is independent, HLA mismatch and antibody production could change due to re-transplantations. The fourth sensitivity analysis excluded such mechanisms by showing similar results (Supplementary Table S2) [18]. In addition to regression coefficients, hazard ratios and their 95% confidence intervals, we report p-values, which were considered statistically significant at p < 0.05. We analyzed the data using IBM SPSS Statistics 27 and R x64 i4.1.3 and R studio 2023.

## Results

**FIGURE 1:**
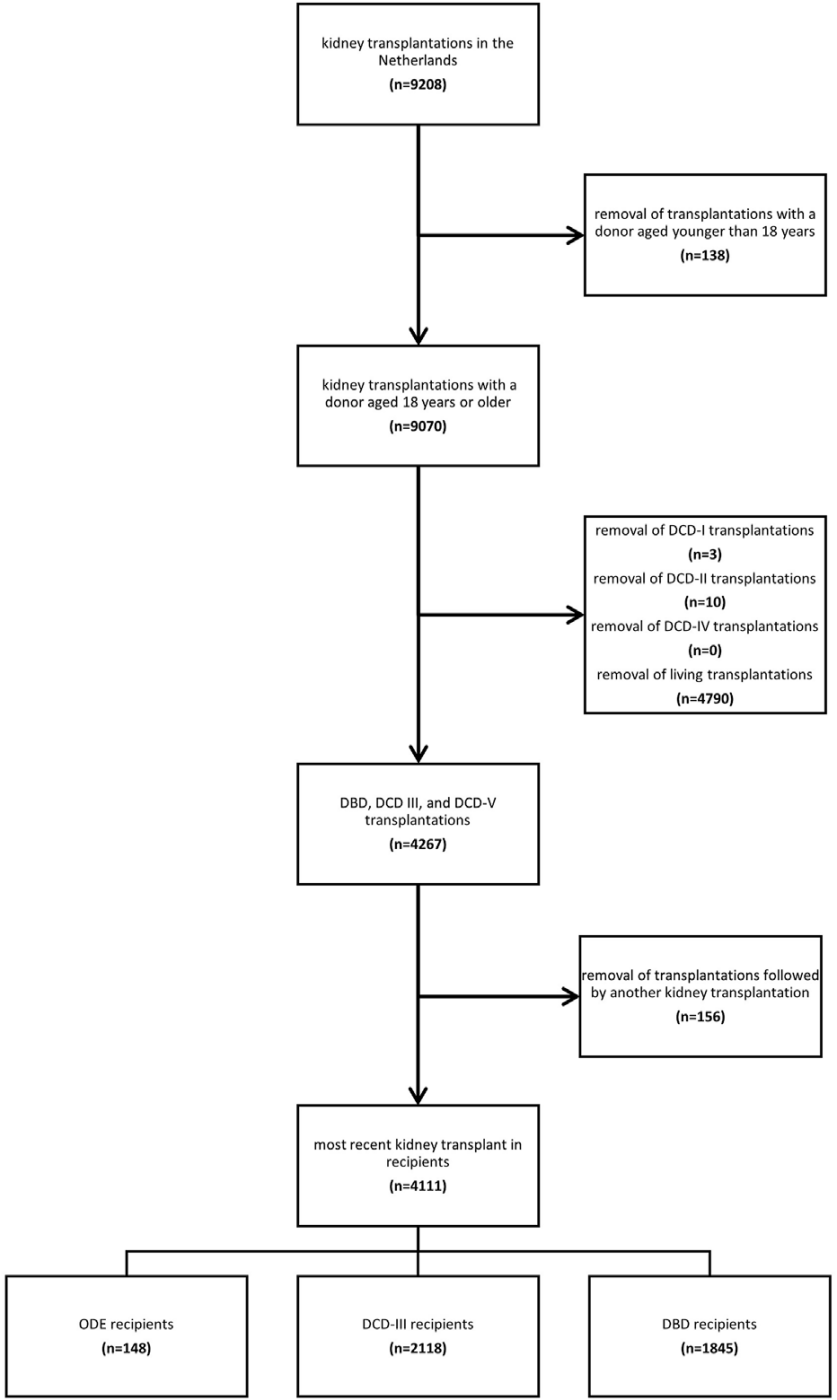
Flowchart of transplantation inclusions and exclusions.

Of the 9,208 kidney transplantations performed in the Netherlands between 1st January 2012 and 31st December 2021, 9,070 were from donors aged 18 years and older. After excluding 4,790 transplantations from living donors and 13 transplantations from DCD-I, DCD-II, and DCD-IV donors, and after excluding previous transplantations within the recipients, 4,111 kidney transplant recipients remained, with kidney transplants originating from 2,730 unique donors (Figure 1). In total, 148 recipients received a kidney from a donor after ODE, 2,118 from a donor after DCD-III, and 1,845 from a donor after DBD (Figure 1).

Donor recipient and transplantation baseline characteristics are presented in Table 1. ODE donors had lower serum creatinine concentrations (p = 0.046) compared to DBD donors. ODE recipients were younger (p = 0.034) than DCD-III recipients. A minor, not clinically relevant difference, was found in WIT between ODE transplantations and DCD-III transplantations (p = 0.022). As expected, WIT was longer in ODE as compared to DBD transplantations (p < 0.001). CIT was shorter in ODE transplantations as compared to DBD (p < 0.001), no difference was found for CIT in ODE and DCD-III (Supplementary Table S3).

**TABLE 1:** Baseline characteristics of donors and recipients, and graft characteristics.

Characteristics	ODE	DCD-III	DBD	p-value
Donor, n	91	1,304	1,335	
Age (yrs)	53 ± 12	55 ± 14	53 ± 14	<0.001^a^
Sex (male, n)	55% (50)	61% (801)	50% (663)	<0.001^b^
Creatinine (µmol/L)	66 ± 17	68 ± 29	77 ± 45	<0.001^a^
Hypertension (n)	14% (11)	27% (301)	29% (228)	0.021^b^
Diabetes (n)	0% (0)	2% (19)	2% (20)	0.512^c^
Smoking (n)	54% (49)	56% (717)	56% (691)	0.896^b^
Smoking pack years	22 ± 15	26 ± 17	25 ± 16	0.197^a^
Recipient, n	148	2,118	1845	
Age (yrs)	55 ± 13	58 ± 13	55 ± 15	<0.001^a^
Sex (male, n)	61% (91)	63% (1,340)	61% (1,129)	0.399^b^
Dialysis time (yrs)	2.7 (1.5–4.6)	2.4 (1.4–3.9)	2.6 (1.4–4.2)	0.001^a^
PRA				<0.001^c^
≤5% (n)	89% (121)	92% (1866)	88% (1,544)	
6%–84% (n)	11% (15)	7% (141)	11% (192)	
≥85% (n)	0% (0)	1% (17)	1% (23)	
Graft, n	148	2,118	1845	
Warm ischemia time (min)^a^	15 (13–18)	16 (13–19)	0 (0–0)	<0.001
Cold ischemia time (hours)^a^	12 ± 5	13 ± 5	15 ± 7	<0.001
Anastomosis time (min)^a^	32 ± 12	33 ± 13	33 ± 15	0.506

^a^ One-Way Anova.

^b^ Chi square test.

^c^ Fisher’s exact test.

ODE, organ donation after euthanasia; DCD, donation after circulatory death; DCD-III, donation after circulatory death following withdrawal of life sustaining treatments in the ICU. DBD, donation after brain death; PRA, panel reactive antibody. Negative ischemia periods and negative anastomosis times have been removed from the set. Cold ischemia times and anastomosis times of zero have been removed from the set. Donor sample sizes of ODE, DCD-III and DBD are, respectively, 91, 1304, 1335. Recipient sample sizes of ODE, DCD-III and DBD are, respectively, 148, 2118, 1845. Graft sample sizes of ODE, DCD-III and DBD are, respectively, 148, 2118, 1845. P-values <0.05 indicate a statistical difference in the overall comparison of donor categories.

Initial graft function was available for ODE 127 out of 148 (86%), for DCD-III 1940 out of 2,118 (92%), and for DBD 1636 out of 1845 kidneys (89%) (Table 2). First-week post-transplantation outcomes showed that immediate graft function was higher in ODE, which was similar to DBD, when compared to DCD-III (overall p-value: p < 0.001) (Table 2). DCD-III showed more delayed graft function as compared to ODE and DBD, whereas primary non-function was similar (overall p-value: p < 0.001) (Table 2; Supplementary Table S1).

**TABLE 2:** Initial graft function and transplant function using estimated glomerular filtration rate (eGFR) up until 10 years after transplantation.

Variable	ODE N = 148	DCD-III N = 2,118	DBD N = 1845	p-value
Initial graft function				<0.001
Immediate function (n) Delayed graft function (n) Primary non-function (n)	76% (96)	47% (904)	73% (1,201)	
	22% (28)	50% (976)	25% (401)	
	2% (3)	3% (60)	2% (34)	
Transplant function over time				
eGFR at 3 months (mL/min)	46 (1.6)	43 (0.6)	46 (0.6)	<0.001
eGFR at 1 year (mL/min)	48 (1.6)	46 (0.4)	48 (0.5)	0.001
eGFR at 2 years (mL/min)	48 (1.9)	46 (0.5)	48 (0.6)	0.092
eGFR at 3 years (mL/min)	50 (2.5)	46 (0.6)	47 (0.7)	0.089
eGFR at 4 years (mL/min)	48 (2.5)	46 (1.0)	47 (0.7)	0.948
eGFR at 5 years (mL/min)	48 (2.4)	46 (0.9)	46 (0.7)	0.791
eGFR at 6 years (mL/min)	50 (3.1)	47 (0.9)	47 (1.0)	0.631
eGFR at 7 years (mL/min)	57 (4.4)	47 (1.0)	46 (1.1)	0.163
eGFR at 8 years (mL/min)	52 (5.4)	48 (1.4)	45 (1.3)	0.305
eGFR at 9 years (mL/min)	59 (10.3)	50 (1.5)	43 (1.8)	0.006
eGFR at 10 years (mL/min)	58 (13.4)	46 (2.1)	46 (3.1)	0.718

Sample sizes of ODE, DCD-III, and DBD, grafts are, respectively, 148, 2118, 1845. Transplant function over time is presented as mean (SE). The number of observations for creatinine of ODE recipients after 3 months, 1 year, 2 years, 3 years, 4 years, 5 years, 6 years, 7 years, 8 years, 9 years, and 10 years were 129, 124, 97, 71, 60, 35, 29, 14, 10, 5, 2, respectively. The number of observations for creatinine of DCD-III recipients after 3 months, 1 year, 2 years, 3 years, 4 years, 5 years, 6 years, 7 years, 8 years, 9 years, and 10 years were 1933, 1823, 1,497, 1,157, 923, 732, 524, 368, 248, 159, 67, respectively. The number of observations for creatinine of DBD, recipients after 3 months, 1 year, 2 years, 3 years, 4 years, 5 years, 6 years, 7 years, 8 years, 9 years, and 10 years were 1,647, 1,500, 1,268, 1,054, 835, 657, 496, 368, 242, 148, 66, respectively. P-values <0.05 indicate a statistical difference in the overall comparison of donor categories.

Mean graft function over time by eGFR is shown in Table 2. Longitudinal mixed-effects regression analyses adjusted for donor category and time (model 1, Table 3) showed that, compared to DBD, longitudinal eGFR for ODE was (β: 95% CI) 1.64 mL/min/1.73 m2 (−1.84; 5.13) and for DCD-III was −1.47 mL/min/1.73m2 (−2.74;-0.20) over the 10-year period. After additional adjustments for donor sex, donor age, donor smoking, recipient age, recipient sex, cold ischemic period, anastomosis time, and initial graft function (model 2, Table 3), and further adjustment for donor hypertension, donor diabetes mellitus, WIT and transplant PRA (model 3, Table 3) this association disappeared. Mixed-effects analyses with random slopes for time showed similar results (model 1–3, Supplementary Table S4).

**TABLE 3:** Longitudinal association between donor categories and estimated glomerular filtration rate over 10 years.

Variable	eGFR β (95% CI)	p-value	p-value ODE vs. DCD-III
Model 1, multivariable			
Donor category			
DBD ODE DCD-III	Reference 1.64 (−1.84; 5.13) −1.47 (−2.74;-0.20)	0.356 0.023	0.078
Model 2, multivariable			
Donor category			
DBD ODE DCD-III	Reference 0.39 (−2.95; 3.72) 0.03 (−1.33; 1.38)	0.821 0.969	0.833
Model 3, multivariable Donor category			
DBD ODE DCD-III	Reference −1.25 (−6.38; 3.88) −0.14 (−3.82; 3.53)	0.634 0.939	0.592

Data are regression coefficients of fixed effects (β) with their 95%CI that indicate the longitudinal association between donor category, and eGFR over a 10-year period, with DBD as reference category. Estimated glomerular filtration rate (eGFR) is the dependent variable in all models. Random intercepts were used for recipient ID. Model 1, with 17,799 observations and 3,599 recipient IDs, includes fixed effects of donor category and time. Model 2, with 14,135 observations and 2,946 recipient IDs, is model 1 additionally adjusted for donor sex, donor age, donor smoking, recipient age, recipient sex, CIT, AT, and initial graft function. Model 3, with 9,385 observations and 1857 recipient IDs, is model 2 additionally adjusted for donor hypertension, donor diabetes, WIT and transplant PRA. Negative coefficients of fixed effects indicate lower eGFR per donor category, as compared to DBD. P-values <0.05 indicate a statistical significant regression coefficient.

After exclusion of primary non-functioning grafts, over a median follow-up period of 4.0 years (IQR 2.0–6.6), 1810 grafts were transplanted, which included 72 ODE, 958 DCD-III, and 780 DBD grafts. Over the median follow-up period, 213 grafts (11.8%) failed. This included 7 grafts (9.7%) for ODE, 93 grafts (9.7%) for DCD-III, and 113 grafts (14.5%) for DBD. Median follow-up periods of each donor category were 3.7 years (IQR 2.0–5.8) for ODE, 4.0 years (IQR 2.0–6.3) for DCD-III and 4.1 years (IQR 2.1–7.0) for DBD grafts.

When studying the association between graft failure and donor category, compared to DBD, the hazard ratio for ODE was (HR: 95% CI) 0.67 (0.33–1.36) and for DCD-III was 0.71 (0.57–0.88) using crude Cox regression analysis (model 1, Table 4). After adjustment for donor sex, donor age, donor smoking, recipient age, recipient sex, cold ischemic period, anastomosis time and initial graft function, the hazard ratio was, compared to DBD, 0.57 (0.25–1.29) for ODE and 0.56 (0.43–0.73) for DCD-III. After additional adjustments for donor hypertension, donor diabetes mellitus, WIT, and transplant PRA, the statistically significant difference between DBD and DCD-III grafts disappeared. The proportional hazards assumption was met (Supplementary Figures S1–S3).

**TABLE 4:** Association between ODE, DCD-III and DBD, and graft failure.

Variable	Graft failure Hazard ratio (95% CI)	p-value	p-value ODE vs. DCD-III
Model 1, crude Donor category			
DBD ODE DCD-III	Reference 0.67 (0.33–1.36) 0.71 (0.57–0.88)	0.266 0.001	0.873
Model 2, multivariable			
Donor category			
DBD ODE DCD-III	Reference 0.57 (0.25–1.29) 0.56 (0.43–0.73)	0.179 <0.001	0.969
Model 3, multivariable Donor category			
DBD ODE DCD-III	Reference 0.24 (0.05–1.11) 0.52 (0.25–1.06)	0.068 0.074	0.287

Data are HR, with their 95%CI that indicate the association between donor category and graft failure, with DBD as reference category. Model 1 crude with 3606 observations and 360 events. Model 2, with 2953 observations and 281 events, adjusted for donor sex, donor age, donor smoking, recipient age, recipient sex, CIT, AT, and initial graft function. Proportional hazard assumption model 2 was met (p 0.221). Model 3, with 1860 observations and 176 events, additionally adjusted for donor hypertension, donor diabetes, WIT, and transplant PRA., Proportional hazard assumption model 3 was met (p = 0.285). HR higher than 1 indicating a higher hazard per donor category, as compared to DBD. P-values <0.05 indicate a statistical significant regression coefficient.

The four sensitivity analyses, re-analyzing the above models by replacing the recipients for organs donated (i.e., including all kidneys transplanted in each recipient; sensitivity analysis 1, Supplementary Table S2); in which recipient death was not censored, but included as an event; (sensitivity analysis 2, Supplementary Table S2); in which primary non-function was included (sensitivity analysis 3, Supplementary Table S2); and in which only the first transplantation of each recipient was included (sensitivity analysis 4, Supplementary Table S2), all showed similar results as the primary analyses.

## Discussion

This study addresses the transplant outcomes of kidneys donated after euthanasia over a 10-year study period, compared to DCD-III and DBD, and has three main findings. First, immediate graft function was higher in ODE, when compared to DCD-III, and similar to DBD. Second, longitudinally, eGFR for ODE did not differ from eGFR for DBD and eGFR for DCD-III over 10 years, after adjustment for donor sex, donor age, donor smoking, recipient age, recipient sex, CIT, AT, initial graft function and donor hypertension, donor diabetes mellitus, WIT and transplant PRA. Third, graft failure for ODE did not differ from graft failure for DBD and graft failure for DCD-III, after adjustment for donor sex, donor age, donor smoking, recipient age, recipient sex, CIT, AT, and initial graft function, donor hypertension, donor diabetes mellitus, WIT and transplant PRA.

Human kidney transplantation remains the treatment of choice for the majority of patients with end-stage renal failure [19–21]. Despite increased numbers of donor organs due to expanded donor criteria, organs from living donors, and donation after circulatory death, the gap between the demand and availability of kidneys for transplantation remains substantial [22–25]. Although the results for ODE kidneys regarding longitudinal eGFR and graft failure were not statistically significantly different compared to those for DBD or DCD-III kidneys, the overall results for ODE support the concept that ODE kidneys are a promising extension of the donor pool. Notably, extension of the donor pool is not the primary goal of the procedure, because ODE is the patient’s final altruistic wish.

Previous research on the outcomes of kidney transplantations following ODE was done in smaller cohorts and case series [11, 12], while no study has assessed longitudinal eGFR and graft function over 10 years. In contrast to others who included data that did not measure an extensive set of potential confounders [18] or only studied ODE compared to DCD-III [14], we focused on DBD, DCD-III, and ODE, using comprehensive data from a nationwide registry.

With regard to other organs donated following ODE, preliminary studies on graft function of transplanted lungs after ODE [26–28] yielded outcome results comparable to DCD-III grafts, and similar results were reported for transplanted livers [29–31]. Recently, the first successful heart transplantation after donation after euthanasia was published [32]. The current study thus found comparable transplant outcomes between ODE and DCD-III [29] and between ODE, DCD-III, and DBD [26] regarding graft failure for kidneys transplanted.

In the future, more patients will request to donate their organs after euthanasia, and it is expected that an increasing number of countries will allow this procedure. Observational data showed that approximately 10% of all patients undergoing euthanasia might be medically eligible to donate at least one organ [33].

However, the vast majority of patients who undergo euthanasia are suffering from end-stage malignancy, which makes them unsuitable as a donor. “Euthanasia donors” often suffer from neurodegenerative or psychiatric disorders, which are not primarily associated with deterioration of organ function of potentially transplantable organs, such as the heart, lungs, liver, kidneys and pancreas [4, 34]. DCD-III donors, who die after withdrawal from life-sustaining therapy, inevitably suffer from hypoxia, hypotension, and inadequate organ perfusion during the progression to circulatory arrest (agonal phase) and the mandatory 5-min period of warm, pulseless ischemia [35, 36]. Donors after brain death suffer from a systemic inflammatory response with a potentially negative impact on graft outcomes [37].

This study has strengths and limitations. A major strength is the large nationwide registry including all donations and transplantations in the Netherlands with a comprehensive set of donor, recipient and transplantation variables that created the opportunity to investigate potential confounding in the associations under investigation. Indeed, different studies have shown associations between either prolonged CIT or prolonged AT or both, and both kidney function and post-transplant graft failure [38, 39]. AT has also been associated with delayed graft function [40]. Hence the adjustments for these variables in the models of the present study. Furthermore, the Cox models were adjusted for transplant PRA as it indicates the risk of transplant failure to the host-response to transplantation [13, 14]. In addition, hypertension and diabetes mellitus, and tobacco exposure, have each been associated to worse eGFR [41]. Therefore, models were adjusted for recipient hypertension and diabetes mellitus and donor smoking behavior. As no information on smoking behavior in recipients was available, residual confounding might have caused us to underestimate the present associations, although most likely recipient smoking behavior is not dependent on ODE, DBD or DCD-III donors [42]. The criteria for HLA-mismatch are different for the first transplantation and for later transplantations and we chose to study the population that comprises the most recent transplantation of recipients in the analyses. Therefore, the associations could not be adjusted for HLA mismatch and this could have led to an underestimation of the present results.

Another strength is a total of four sensitivity analyses that were conducted. In sensitivity analysis 1, all kidney transplantations within recipients were included to investigate whether including multiple transplants for the same recipients changed the results. In the second sensitivity analysis, recipient death with functioning transplant, was included as an event, as graft survival and recipient deaths may be related. In sensitivity analysis 3, primary non-function was included as an event, despite it being considered a short-term transplant outcome. The fourth sensitivity analysis, considering only the first kidney transplantation per recipient, was performed as HLA matching is not independent of the number of transplantations within a recipient. The presence of donor-specific HLA antibodies before transplantation is considered a risk factor for graft rejection. Furthermore, waiting time until transplantation increases the risk of higher sensitization levels. Organ transplantation induces HLA alloimmunization, affecting the matching of a re-transplant and waiting time until transplantation [18].

Another strength lies in the approach of investigating the 10-year post-transplantation outcome in two different ways: using Cox proportional hazards analyses for the association between donor categories and graft failure and longitudinal analyses for the association between donor categories and eGFR, which together increase the validity of the results. The study has several limitations as well. In the Netherlands, post-mortem donation allocation is based on blood and tissue match between the donor and recipient of the organ, the medical urgency of the recipient, and other circumstances related to the condition of the organ or the background of the recipient. Neither the donor nor their relatives are allowed to choose a recipient [43]. One donor could have donated two kidneys to two different recipients. However, we did not take into account the potential dependency between recipients who received a kidney from the same donor, which is a limitation of this study. This dependency between recipients could potentially have affected transplant outcomes, although the direction of its effect is difficult to assess. With regard to sensitivity analysis 1, to investigate whether including multiple transplants for the same recipients changed the results, it needs to be recognized that multiple kidney transplantations within a recipient during the disease course are dependent and this was not accounted for in the Cox models.

Furthermore, the current study’s ODE sample size decreased considerably after 6 years of follow-up, potentially compromising the reliability of the longer-term findings. This limitation requires a cautious interpretation of results during the extended follow-up period of ODE grafts, suggesting that conclusions towards 10 years should be less strongly conveyed. Future research should thus focus on larger cohorts to enhance the robustness of long-term conclusions. Given the contemporary annual increase in the number of ODE procedures, it is, however, estimated that an analysis of the first 300 kidney transplantations will take at least 5 additional years.

Another limitation of the study is that recipient ethnicity could not be used in the MDRD4 equation, due to lack of information. This has potentially led to a small underestimation of follow-up eGFR. However, since the same error has been made within each recipient, this will not make major difference in the trend over time.

In conclusion, kidneys transplanted after euthanasia have a good immediate graft function and a comparable longitudinal eGFR over 10 years and comparable hazard for graft failure when compared to kidneys transplanted after brain death or circulatory arrest. Overall, these results support the concept that ODE kidneys are a promising contribution to the donor pool, and ODE should be continued.

## Data Availability

The data analyzed in this study is subject to the following licenses/restrictions: The dataset is available upon specific request to the NTS (Netherlands Transplantation Society). Requests to access these datasets should be directed to Cynthia Konijn-Janssen, dataverzoek@transplantatiestichting.nl.

## References

[1] Kootstra G Daemen JH Oomen AP. Categories of Non-Heart-Beating Donors. Transpl Proc (1995) 27(5):2893–4. 7482956

[2] Kootstra G van Heurn E. Non-Heartbeating Donation of Kidneys for Transplantation. Nat Clin Pract Nephrol (2007) 3(3):154–63. 10.1038/ncpneph0426 17322927

[3] Detry O Le Dinh H Noterdaeme T De Roover A Honore P Squifflet JP Categories of Donation After Cardiocirculatory Death. Transpl Proc (2012) 44(5):1189–95. 10.1016/j.transproceed.2012.05.001 22663982

[4] Bollen J de Jongh W Hagenaars J van Dijk G ten Hoopen R Ysebaert D Organ Donation After Euthanasia: A Dutch Practical Manual. Am J Transplant (2016) 16(7):1967–72. 10.1111/ajt.13746 26842128

[5] Silva ESV Silva AR Rochon A Lotherington K Hornby L Wind T Organ Donation Following Medical Assistance in Dying, Part II: A Scoping Review of Existing Processes and Procedures. JBI Evid Synth (2024) 22(2):195–233. 10.11124/jbies-22-00140 37489247 PMC10871582

[6] Weiss MJ Dupras-Langlais M Lavigne MJ Lavigne S Martel AC Chaudhury P. Organ Donation After Medical Assistance in Dying: A Descriptive Study From 2018 to 2022 in Quebec. CMAJ (2024) 196(3):E79–E84. 10.1503/cmaj.230883 38286494 PMC10833101

[7] Allard J Fortin MC. Organ Donation After Medical Assistance in Dying or Cessation of Life-Sustaining Treatment Requested by Conscious Patients: The Canadian Context. J Med Ethics (2017) 43(9):601–5. 10.1136/medethics-2016-103460 28031256

[8] Bollen J Ten Hoopen R Ysebaert D van Mook W van Heurn E. Legal and Ethical Aspects of Organ Donation After Euthanasia in Belgium and the Netherlands. J Med Ethics (2016) 42(8):486–9. 10.1136/medethics-2015-102898 27012736

[9] van Dijk N Stärcke P de Jongh W Jansen N Shaw D Bollen J Organ Donation After Euthanasia in Patients Suffering From Psychiatric Disorders: 10-Years of Preliminary Experiences in the Netherlands. Transpl Int (2023) 9(36):10934–2277. 10.3389/ti.2023.10934 PMC9948004 36846601

[10] Silva ESV Silva A Rochon A Lotherington K Hornby L Wind T Outcomes from Organ Donation Following Medical Assistance in Dying: A Scoping Review. Transpl Rev (Orlando) (2023) 37(1):100748. 10.1016/j.trre.2023.100748 36774782

[11] Luke PP Skaro A Sener A Tang E Levine M Sami S Kidney Transplant Outcomes after Medical Assistance in Dying. Can Urol Assoc J. (2022) 16(2):E108-E110. 10.5489/cuaj.7304 34582335 PMC8932416

[12] Bollen J Snoeijs M Ten Hoopen R Shaw D van Mook W van Heurn E Promising Results of Kidney Transplantation From Donors Following Euthanasia. Transplantation (2020) 104(S294):S394–1193. 10.1097/01.tp.0000700584.87933.96

[13] Lim WH Chapman JR Wong G. Peak Panel Reactive Antibody, Cancer, Graft, and Patient Outcomes in Kidney Transplant Recipients. Transplantation (2015) 99(5):1043–50. 10.1097/TP.0000000000000469 25539466

[14] Nwakanma LU Williams JA Weiss ES Russell SD Baumgartner WA Conte JV. Influence of Pretransplant Panel-Reactive Antibody on Outcomes in 8,160 Heart Transplant Recipients in Recent Era. The Ann Thorac Surg (2007) 84(5):1556–62. 10.1016/j.athoracsur.2007.05.095 17954062

[15] Halazun KJ Al-Mukhtar A Aldouri A Willis S Ahmad N. Warm Ischemia in Transplantation: Search for a Consensus Definition. Transpl Proc (2007) 39(5):1329–31. 10.1016/j.transproceed.2007.02.061 17580133

[16] Levey AS Coresh J Fau - Greene T Greene T Fau - Stevens LA Stevens La Fau - Zhang YL Using Standardized Serum Creatinine Values in the Modification of Diet in Renal Disease Study Equation for Estimating Glomerular Filtration Rate. Ann Intern Med (2006) 145(4):247–54. 10.7326/0003-4819-145-4-200608150-00004 16908915

[17] von Elm E Altman DG Egger M Pocock SJ Gøtzsche PC Vandenbroucke JP The Strengthening the Reporting of Observational Studies in Epidemiology (STROBE) Statement: Guidelines for Reporting Observational Studies. The Lancet (2007) 370(9596):1453–7. 10.1016/S0140-6736(07)61602-X 18064739

[18] Hyun J Park K Fau - Yoo Y Yoo Y Fau - Lee B Fau - Han By LB Effects of Different Sensitization Events on HLA Alloimmunization in Solid Organ Transplantation Patients. Transpl Proc. (2012) 44(1):222–5. 10.1016/j.transproceed.2011.12.049 22310619

[19] Purnell TS Auguste P Crews DC Lamprea-Montealegre J Olufade T Greer R Comparison of Life Participation Activities Among Adults Treated by Hemodialysis, Peritoneal Dialysis, and Kidney Transplantation: A Systematic Review. Am J Kidney Dis (2013) 62(5):953–73. 10.1053/j.ajkd.2013.03.022 23725972 PMC3809150

[20] Wolfe RA Ashby VB Milford EL Ojo AO Ettenger RE Agodoa LY Comparison of Mortality in All Patients on Dialysis, Patients on Dialysis Awaiting Transplantation, and Recipients of a First Cadaveric Transplant. N Engl J Med (1999) 341(23):1725–30. 10.1056/NEJM199912023412303 10580071

[21] Azegami T Kounoue N Sofue T Yazawa M Tsujita M Masutani K Efficacy of Pre-emptive Kidney Transplantation for Adults With End-Stage Kidney Disease: A Systematic Review and Meta-Analysis. Ren Fail (2023) 45(1):2169618. 10.1080/0886022X.2023.2169618 36705051 PMC9888453

[22] Tullius SG Rabb H. Improving the Supply and Quality of Deceased-Donor Organs for Transplantation. N Engl J Med (2018) 378(20):1920–9. 10.1056/NEJMra1507080 29768153

[23] Snoeijs MG Schaubel DE Hene R Hoitsma AJ Idu MM Ijzermans JN Kidneys from Donors after Cardiac Death Provide Survival Benefit. J Am Soc Nephrol (2010) 21(6):1015–21. 10.1681/ASN.2009121203 20488954 PMC2900965

[24] Active Kidney Waiting List in 2023 2024. Available from: https://statistics.eurotransplant.org/index.php?search_type=waiting+list&search_organ=&search_region=All+ET&search_period=by+year&search_characteristic=&search_text. (Accessed April 3, 2024).

[25] Waiting List Mortality in 2023. Available from: https://statistics.eurotransplant.org/index.php?search_type=WL+removals&search_organ=kidney&search_region=All+ET&search_period=2023&search_characteristic=&search_text=&search_collection=. (Accessed April 3, 2024).

[26] Ceulemans LJ Vanluyten C Monbaliu D Schotsmans P Fieuws S Vandervelde CM Lung Transplant Outcome Following Donation After Euthanasia. J Heart Lung Transpl (2022) 41(6):745–54. 10.1016/j.healun.2022.01.1375 35227627

[27] Watanabe T Kawashima M Kohno M Yeung J Downar J Healey A Outcomes of Lung Transplantation from Organ Donation after Medical Assistance in Dying: First North American Experience. Am J Transpl (2022) 22(6):1637–45. 10.1111/ajt.16971 35108446

[28] Healey A Cypel M Pyle H Mills C Heffren J Katz D Lung Donation After Medical Assistance in Dying at Home. Am J Transpl (2021) 21(1):415–8. 10.1111/ajt.16267 32803817

[29] van Reeven M Gilbo N Monbaliu D van Leeuwen OB Porte RJ Ysebaert D Evaluation of Liver Graft Donation After Euthanasia. JAMA Surg (2020) 155(10):917–24. 10.1001/jamasurg.2020.2479 32777007 PMC7407314

[30] Ray S Torres-Hernandez A Bleszynski MS Parmentier C McGilvray I Sayed BA Medical Assistance in Dying (MAiD) as a Source of Liver Grafts: Honouring the Ultimate Gift. Ann Surg (2023) 277(5):713–8. 10.1097/SLA.0000000000005775 36515405

[31] Glinka J Sachar Y Tang E Brahmania M Hwang J Waugh E Liver Transplantation With Donation After Medical Assistance in Dying: Case Series and Systematic Review of the Literature. Liver Transpl (2023) 29(6):618–25. 10.1097/LVT.0000000000000100 36896964

[32] Tchana-Sato V Hans G Brouckaert J Detry O Van Cleemput J Rex S Successful Heart Transplantation From Donation after Euthanasia with Distant Procurement Using Normothermic Regional Perfusion and Cold Storage. Am J Transpl (2022) 22(12):3146–9. 10.1111/ajt.17204 36131641

[33] Bollen J van Smaalen T Ten Hoopen R van Heurn E Ysebaert D van Mook W. Potential Number of Organ Donors After Euthanasia in Belgium. JAMA (2017) 317(14):1476–7. 10.1001/jama.2017.0729 28399240

[34] van Dijk N Starcke P de Jongh W Jansen N Shaw D Bollen J Organ Donation After Euthanasia in Patients Suffering from Psychiatric Disorders: 10-Years of Preliminary Experiences in the Netherlands. Transpl Int (2023) 36:10934. 10.3389/ti.2023.10934 36846601 PMC9948004

[35] Domínguez-Gil BA-O Ascher N Capron AM Gardiner D Manara AR Bernat JL Expanding Controlled Donation After the Circulatory Determination of Death: Statement from an International Collaborative. Intensive Care Med (2021) 47(3):265–281. 10.1007/s00134-020-06341-7 33635355 PMC7907666

[36] Hyun SA-O Haam SA-O. Donation After Circulatory Death in Lung Transplantation. J Chest Surg (2022) 55(4):283–287. 10.5090/jcs.22.060 35924534 PMC9358165

[37] Morrissey PE Monaco AP. Donation After Circulatory Death: Current Practices, Ongoing Challenges, and Potential Improvements. Transplantation (2014) 97(3):258–64. 10.1097/01.TP.0000437178.48174.db 24492420

[38] Debout A Foucher Y Trébern-Launay K Legendre C Kreis H Mourad G Each Additional Hour of Cold Ischemia Time Significantly Increases the Risk of Graft Failure and Mortality Following Renal Transplantation. Kidney Int (2015) 87(2):343–9. 10.1038/ki.2014.304 25229341

[39] Foley MA-O Vinson AA-O Skinner TAA Kiberd BA Tennankore KA-O. The Impact of Combined Warm and Cold Ischemia Time on Post-transplant Outcomes. Can J Kidney Health Dis (2023) 10:20543581231178960. 10.1177/20543581231178960 37333478 PMC10272701

[40] Tennankore KK Kim SJ Alwayn IP Kiberd BA. Prolonged Warm Ischemia Time Is Associated with Graft Failure and Mortality After Kidney Transplantation. Kidney Int (2016) 89(3):648–58. 10.1016/j.kint.2015.09.002 26880458

[41] Fu YC Xu ZL Zhao MY Xu K. The Association Between Smoking and Renal Function in People Over 20 Years Old. Front Med (Lausanne) (2022) 9:2296–858X. (Print). 10.3389/fmed.2022.870278 PMC9205397 35721101

[42] Eid HA-OX Moazen EA-O Elhussini M Shoman H Hassan AA-O Elsheikh AA-O The Influence of Smoking on Renal Functions Among Apparently Healthy Smokers. J Multidiscip Healthc (2022) 15:2969–2978. 10.2147/JMDH.S392848 36582586 PMC9793780

[43] Wet op de Orgaandonatie 2022. Available from: https://wetten.overheid.nl/BWBR0008066/2022-01-01/#Hoofdstuk3. (Accessed March 23, 2024).

