# Supplementary material for: Promising Results of Kidney Transplantation From Donors Following Euthanasia During 10-Year Follow-Up: A Nationwide Cohort Study
Source: Transpl Int. 2024 Oct 18;37:13142. doi: 10.3389/ti.2024.13142 (PMC11528710; doi:10.3389/ti.2024.13142)

Supplement tables

Supplemental table 1 Graft fail descriptions and frequencies.

Graft fail description	ODE (n=33) (%)	DCD-III (n=668) (%)	DBD (n=596) (%)	Total (n=1297) (%)
Hyperacute rejection	1 (3.0%)	4 (0.6%)	2 (0.3%)	7 (0.5%)
Infection (not graft related)	1 (3.0%)	10 (1.5%)	12 (2.0%)	23 (1.8%)
Infection of graft	0 (0.0%)	4 (0.6%)	3 (0.5%)	7 (0.5%))
Non-viable kidney	0 (0.0%)	1 (0.1%)	3 (0.5%)	4 (0.3%)
Other (renal)	2 (6.1%)	30 (4.5%)	19 (3.2%)	51 (3.9%)
Patient died with functioning transplant	19 (57.6%)	427 (63.9%)	333 (55.9%)	779 (60.1%)
Permanent non-function	2 (6.1%)	42 (6.3%)	24 (4.0%)	68 (5.2%)
Recurrent primary renal disease	0 (0.0%)	6 (0.9%)	7 (1.2%)	13 (1.0%)
Rejection after stopping all immunosuppressive drugs	1 (3.0%)	2 (0.3%)	5 (0.8%)	8 (0.6%)
Rejection while taking immunosuppressive drugs (acute / chronic)	6 (18.2%)	104 (15.6%)	150 (25.2%)	260 (20.0%)
Removal of functioning graft	0 (0.0%)	4 (0.6%)	2 (0.3%)	6 (0.5%)
Technical problems	0 (0.0%)	1 (0.1%)	0 (0.0%)	1 (0.1%)
Thrombosis / infarction	1 (3.0%)	10 (1.5%)	10 (1.7%)	21 (1.6%)
Unknown	0 (0.0%)	14 (2.1%)	16 (2.7%)	30 (2.3%)
Vascular or ureteric problems	0 (0.0%)	2 (0.3%)	6 (1.0%)	8 (0.6%)
Vascular problems: not operative or rejection related	0 (0.0%)	7 (1.0%)	4 (0.7%)	11 (0.8%)
Total (%)	33 (100%)	668 (100%)	596 (100%)	1297 (100%)

Supplemental Table 2 Association between ODE, DCD-III and DBD, and graft failure, four sensitivity analyses

Variable	Graft failure<br>Hazard Ratio (95% CI)	p-value	p-value<br>ODE vs DCD-III
Sensitivity analyses 1, including 156 transplantations followed by another kidney transplantation, totalling 4267 transplantations within 4111 recipients.			
Model 1, crude			
Donor category			
DBD	Reference		
ODE	0.66 (0.34-1.29)	0.223	
DCD-III	0.81 (0.67-0.98)	0.033	0.546
Model 2, multivariable			
Donor category			
DBD	Reference		
ODE	0.60 (0.28-1.28)	0.188	
DCD-III	0.68 (0.54-0.86)	0.001	0.758
Sensitivity analyses 2, including recipient death as graft failure			
Model 1, crude			
Donor category			
DBD	Reference		
ODE	0.86 (0.58-1.27)	0.437	
DCD-III	1.01 (0.90-1.14)	0.849	0.402
Model 2, multivariable			
Donor category			
DBD	Reference		
ODE	0.87 (0.57-1.33)	0.515	
DCD-III	0.86 (0.74-0.99)	0.037	0.945
Sensitivity analyses 3, including primary non-function			
Model 1, crude			
Donor category			
DBD	Reference		
ODE	0.81 (0.47-1.39)	0.446	
DCD-III	0.82 (0.69-0.98)	0.029	0.960
Model 2, multivariable			
Donor category			
DBD	Reference		
ODE	0.54 (0.26-1.11)	0.092	
DCD-III	0.64 (0.51-0.81)	<0.001	0.626

Sensitivity analyses 4, first transplantation of each recipient, including 3521 transplantations			
Model 1, crude			
Donor category			
DBD	Reference		
ODE	0.61 (0.30-1.24)	0.172	
DCD-III	0.81 (0.66-0.98)	0.030	0.442
Model 2, multivariable			
Donor category			
DBD	Reference		
ODE	0.54 (0.24-1.22)	0.140	
DCD-III	0.68 (0.53-0.86)	0.001	0.591

Four sensitivity analyses. Data are HR with their 95%CI that indicate the association between donor category and mid-term graft failure, with DBD as reference category. The models 1 were crude, and the models 2 were multivariable, adjusted for donor sex, donor age, donor smoking, recipient age, recipient sex, CIT, AT, and initial graft function. Sensitivity analysis 1, including previous transplantations of recipients (n=156, totalling 4267 transplantations of 4111 patients). The number of observations and number of events of model 1 and model 2 were, respectively 3679 observations and 431 events, and 3007 observations and 335 events. Sensitivity analysis 2, with event defined as graft failure, including recipient death. The number of observations and number of events of model 1 and model 2 were, respectively, 3606 observations and 1082 events, and 2953 observations and 863 events. Sensitivity analysis 3, including primary non-function. The number of observations and number of events of model 1 and model 2 were, respectively 3962 observations and 518 events, and 3039 observations and 356 events. Sensitivity analysis 4, including only the first transplantation per recipient. The number of observations and number of events of model 1 and model 2 were, respectively 3521 observations and 415 events, and 2871 observations and 321 events. HR higher than 1 indicating a higher hazard per donor category, as compared to DBD. P-values <0.05 indicate a statistical significant regression coefficient.

Supplemental table 3 Pairwise comparisons of baseline characteristics using Bonferroni post-hoc testing.

	Donor category contrast	Mean difference	p-value
**Donor**			
Age	ODE – DBD	0.4	1.000
	ODE – (DCD-III)	-1.8	0.672
	DBD – (DCD-III)	-2.2	<0.001
Creatinine	ODE – DBD	-10.4	0.046
	ODE – (DCD-III)	-2.0	1.000
	DBD – (DCD-III)	8.4	<0.001
**Recipient**			
Age	ODE – DBD	0.2	1.000
	ODE – (DCD-III)	-3.0	0.034
	DBD – (DCD-III)	-3.1	<0.001
Dialysis time	ODE – DBD	0.2	1.000
	ODE – (DCD-III)	0.5	0.131
	DBD – (DCD-III)	0.3	0.003
**Graft**			
Warm ischemia time	ODE - DBD	15	<0.001
	ODE – (DCD-III)	-1	0.022
	DBD – (DCD-III)	16	<0.001
Cold ischemia time	ODE - DBD	-2.5	<0.001
	ODE – (DCD-III)	-0.3	1.000
	DBD – (DCD-III)	2.2	<0.001

Pairwise comparisons of baseline characteristics using Bonferroni post-hoc testing. Donor sample sizes of ODE, DCD-III and DBD are, respectively, 91, 1304, 1335. Recipient sample sizes of ODE, DCD-III and DBD are, respectively, 148, 2118, 1845. Graft sample sizes of ODE, DCD-III and DBD are, respectively, 148, 2118, 1845. P-values <0.05 indicate a statistical difference in mean between donor categories.

Supplemental Table 4 Longitudinal association between donor categories and estimated glomerular filtration rate over 10 years, with random slopes of time.

**Variable**	**B (95% CI)**	**p-value**	**p-value ODE vs DCD-III**
Model 1, multivariable			
Donor category			
DBD	Reference		
ODE	1.40 (-2.10;4.90)	0.434	
DCD-III	-1.81 (-3.09;-0.53)	0.005	0.071
Model 2, multivariable			
Donor category			
DBD	Reference		
ODE	0.53 (-2.81;3.88)	0.755	
DCD-III	0.14 (-1.22;1.49)	0.841	0.817
Model 3, multivariable			
Donor category			
DBD	Reference		
ODE	-1.18 (-6.29;3.93)	0.653	
DCD-III	-0.16 (-3.82;3.50)	0.932	0.620

Data are regression coefficients of fixed effects (β) with their 95%CI that indicate the longitudinal association between donor category, and eGFR over a 10-year period, with DBD as reference category. The eGFR is the dependent variable in all models. Random intercepts were used for recipient ID and random slopes were used for time. Model 1, with 17799 observations and 3599 recipient IDs, includes donor category. Model 2, with 14135 observations and 2946 recipient IDs, is model 1 additionally adjusted for donor sex, donor age, donor smoking, recipient age, recipient sex, CIT, AT, and initial graft function. Model 3, with 9385 observations and 1857 recipient IDs, is model 2 additionally adjusted for donor hypertension, donor diabetes, WIT and transplant PRA. Negative coefficients of fixed effects indicate lower eGFR per donor category, as compared to DBD. P-values <0.05 indicate a statistical significant regression coefficient.

Supplemental table 5 Pairwise comparisons between donor categories using Chi square Bonferroni post hoc testing.

			Donor category		
			ODE	DCD-III	DBD
Donor sex	Male	Residuals	-0.1	6.0	-6.0
		P-value	1.000	<0.001	<0.001
	Female	Residuals	0.1	-6.0	6.0
		P-value	1.000	<0.001	<0.001
Donor hypertension	No	Residuals	2.6	0.3	-1.4
		P-value	0.055	1.000	1.000
	Yes	Residuals	-2.6	-0.3	1.4
		P-value	0.055	1.000	1.000
Recipient PRA	≤5%	Residuals	-0.4	4.5	-4.4
		P-value	1.000	<0.001	<0.001
	6-84%	Residuals	0.9	-4.4	4.0
		P-value	1.000	<0.001	<0.001
	≥85%	Residuals	-1.2	-1.2	1.6
		P-value	1.000	1.000	0.962
Initial graft function	Immediate function	Residuals	3.8	-16.7	15.4
		P-value	0.001	<0.001	<0.001
	Delayed graft function	Residuals	-3.8	16.3	-15.0
		P-value	0.002	<0.001	<0.001
	Primary non-function	Residuals	-0.2	1.9	-1.8
		P-value	1.000	0.527	0.599

Pairwise comparisons between donor categories using Chi square Bonferroni post hoc testing. Donor sample sizes of ODE, DCD-III and DBD are, respectively, 91, 1304, 1335. Recipient sample sizes of ODE, DCD-III and DBD are, respectively, 148, 2118, 1845. P-value <0.05 indicate a statistical difference between expected proportion and observed proportion in that donor category.

Supplemental table 6 Pairwise comparisons of eGFR between donor categories using Bonferroni post hoc testing.

	Donor category contrast	Mean difference	p-value
eGFR at 3 months	ODE – DBD	0.1	1.000
	ODE – (DCD-III)	3.3	0.396
	DBD – (DCD-III)	3.2	<0.001
eGFR at 1 year	ODE – DBD	-0.4	1.000
	ODE – (DCD-III)	2.1	0.735
	DBD – (DCD-III)	2.4	0.001
eGFR at 9 years	ODE – DBD	16.1	0.252
	ODE – (DCD-III)	9.3	0.952
	DBD – (DCD-III)	-6.8	0.011

Pairwise comparisons of eGFR between donor categories using Bonferroni post hoc testing. Number of observations for eGFR of ODE recipients after 3 months, 1 year and 9 years were, respectively, 129, 124, 5. Number of observations for eGFR of DCD-III recipients after 3 months, 1 year and 9 years were, respectively, 1933, 1823, 159. Number of observations for eGFR of DBD recipients after 3 months, 1 year and 9 years were, respectively, 1647, 1500, 148. P-values <0.05 indicate a statistical difference in mean between donor categories.

Supplemental figure 1 Schoenfeld residuals plots for Cox main model 1 (crude), with 3606 observations and 360 events.

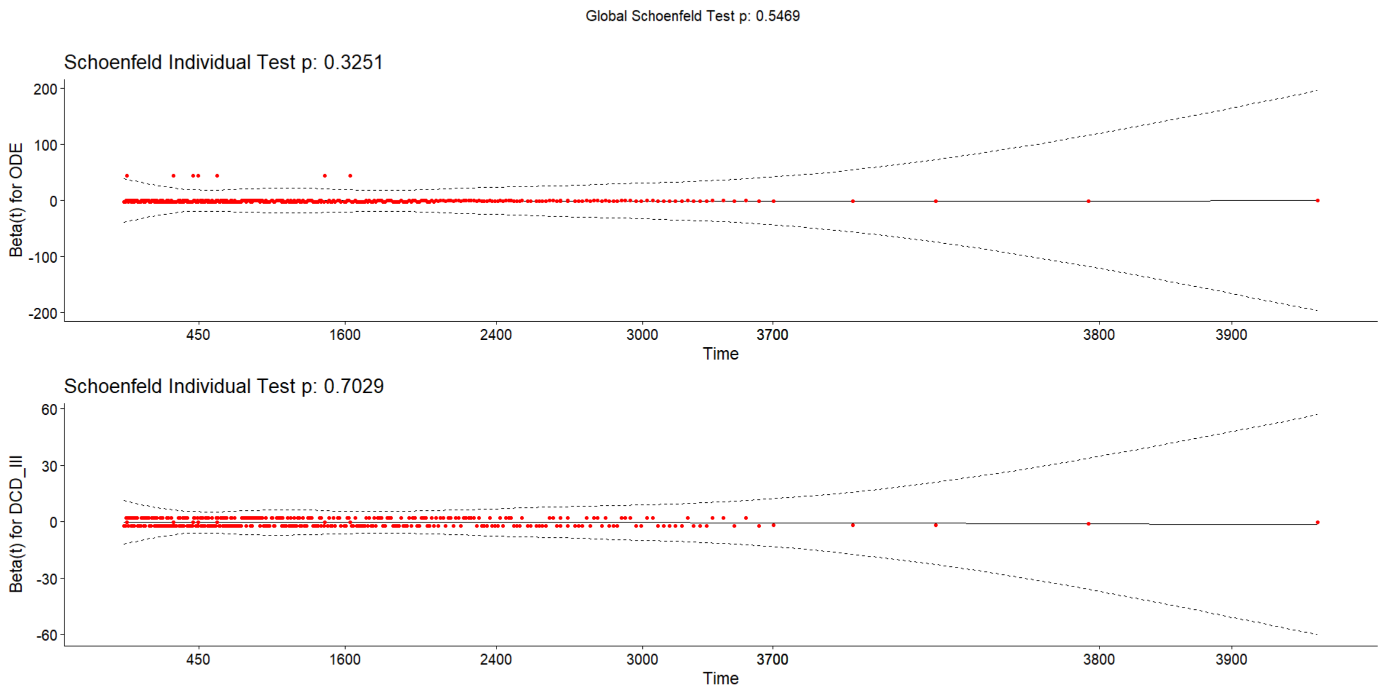

Supplemental figure 2 Schoenfeld residuals plots for Cox main model 2, with 2953 observations and 281 events, adjusted for donor age, donor sex, donor smoking, recipient age, recipient sex, cold ischemic period, anastomosis time and initial graft function.

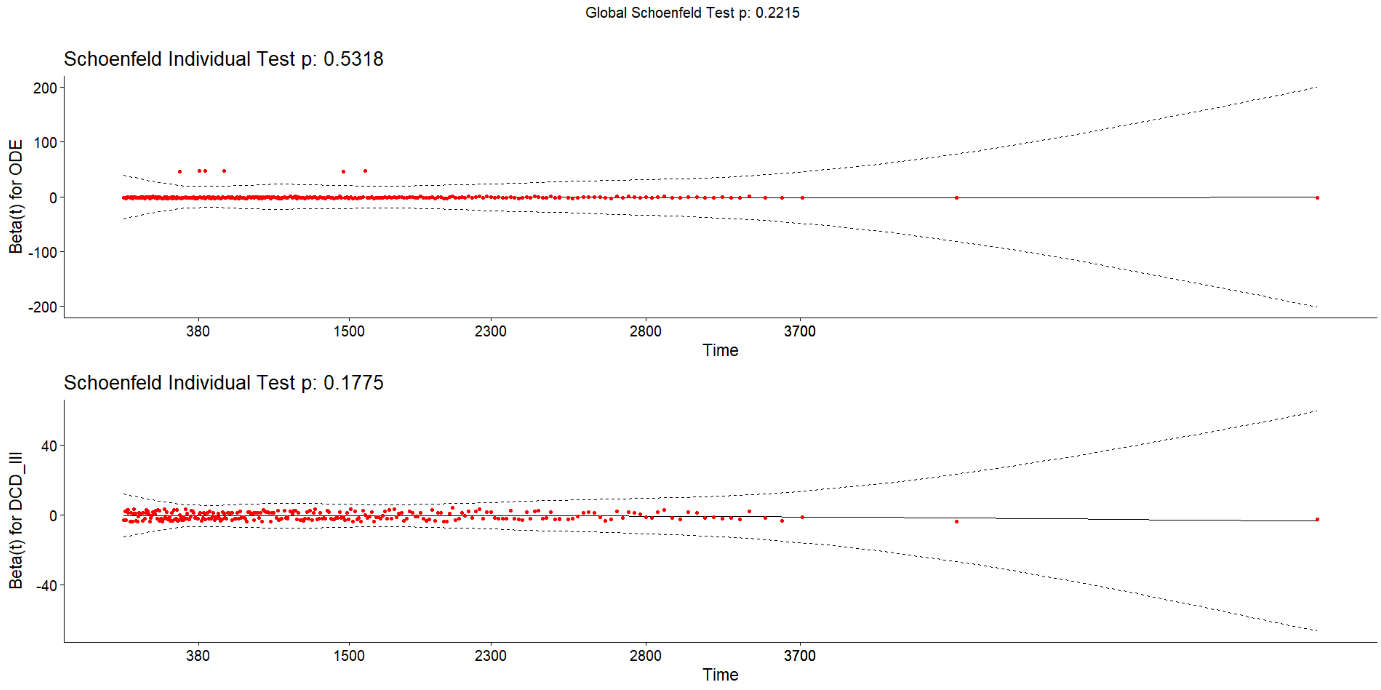

Supplemental figure 3 Schoenfeld residuals plots for Cox main model 3, with 1860 observations and 176 events, adjusted for donor age, donor sex, donor smoking, recipient age, recipient sex, cold ischemic time, anastomosis time, initial graft function, donor hypertension, donor diabetes, warm ischemic time and transplant panel reactive antibodies.

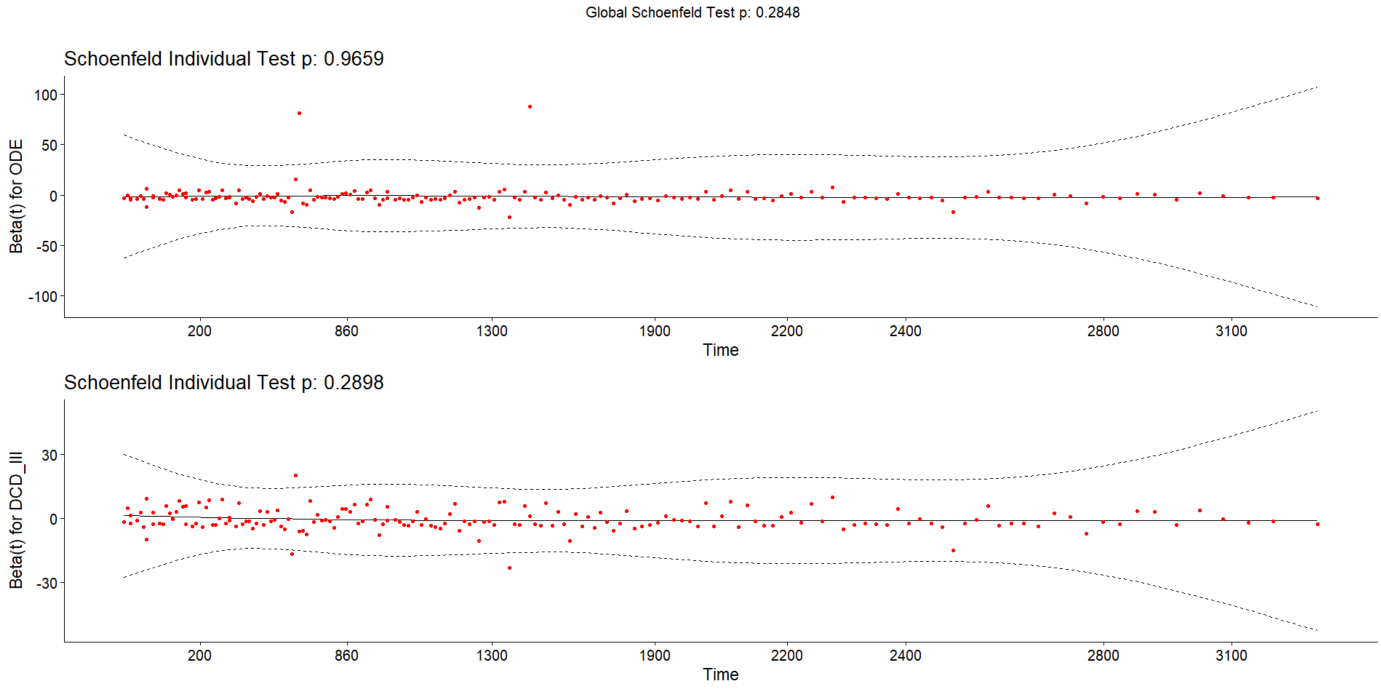

Supplemental figure 4 Plot showing eGFR values and means per donor category over time.

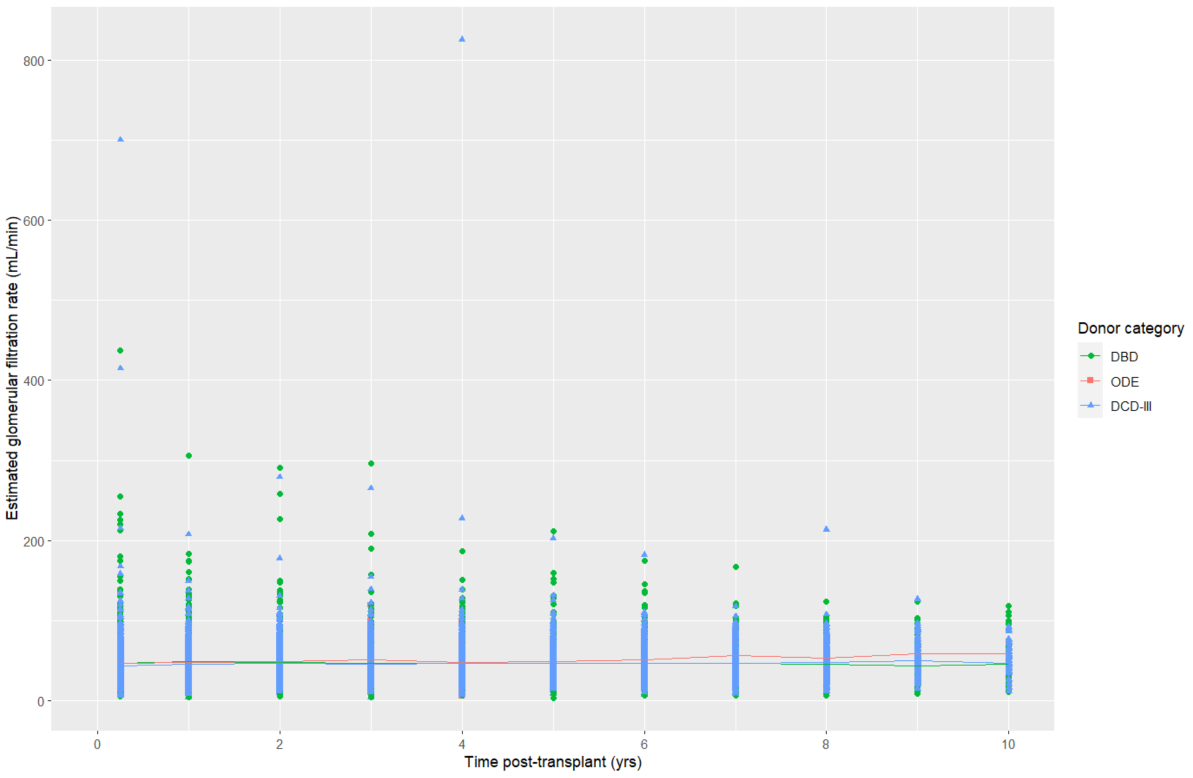

Supplemental figure 5 Plot showing mean eGFR per donor category over time with confidence intervals.

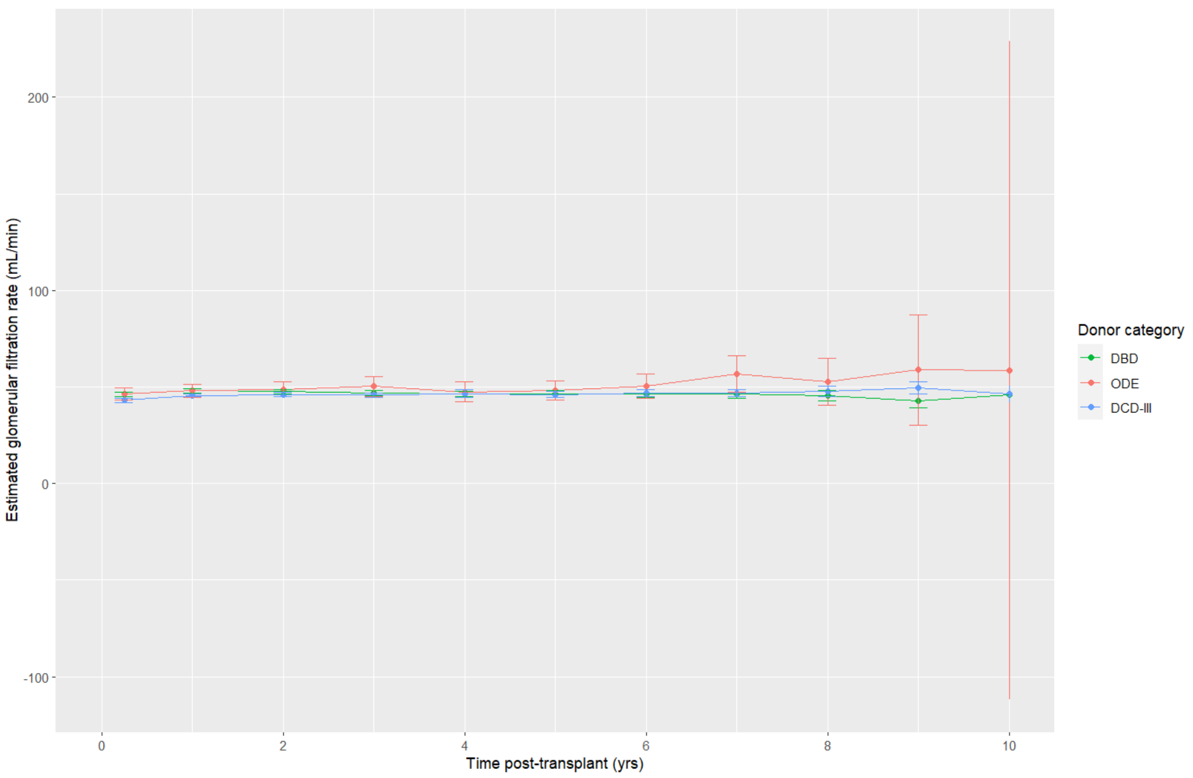

Supplement: Supplementary file 1 [file DataSheet1.pdf]
